# Surgical Outcomes of Adults with Spinal Caries from 1992 to 2019: A Single-Center Study-Risk Factors for the Progression of Kyphosis after Anterior Spinal Fixation Reveal Cases Needing Additional Posterior Instrumentation

**DOI:** 10.3390/jcm13133803

**Published:** 2024-06-28

**Authors:** Mitsuru Furukawa, Kanehiro Fujiyoshi, Takahiro Kitagawa, Reo Shibata, Shogo Hashimoto, Yoshiomi Kobayashi, Tsunehiko Konomi, Yoshiyuki Yato

**Affiliations:** Department of Orthopaedic Surgery, Murayama Medical Center, National Hospital Organization, Tokyo 208-0011, Japankonomitsunehiko@gmail.com (T.K.);

**Keywords:** spinal tuberculosis, paralysis, bone fusion, kyphosis, anterior spinal fusion, posterior spinal fusion

## Abstract

**Background**: This study aims to investigate the postoperative improvement of paralysis, fusion rate and risk factors for kyphosis progression in adults affected with spinal caries. **Methods**: Overall, 134 patients with spinal caries from the thoracic to lumbar spine from 1992 to 2021 were included in this study. Data concerning the affected level (thoracic, thoracolumbar, lumbar, and lumbosacral), bone fusion rate, and progression of the postoperative local kyphosis angle were collected. The risk factors for the progression of local kyphosis angle after anterior spinal fixation (ASF) were determined using linear regression analysis. **Results**: Preoperatively, the degree of spinal cord paralysis was D and E on Frankel classification. Improvement of paralysis was good with surgery, especially from C, D. The overall bone fusion rate was 83.2%. The only factor influencing the progression of local kyphosis angle after ASF was the level of the affected vertebra. Progression of kyphosis angle after ASF was very advanced in the thoracolumbar transition area. **Conclusions**: Surgical improvement in paraplegia and the fusion rate of ASF with only grafted bone was good. However, in patients affected in the thoracolumbar spine region, posterior instrumentation is desirable because of local kyphosis progression risk after surgery.

## 1. Background

Tuberculosis (TB) is a communicable disease that is a major cause of ill health, one of the top ten causes of death worldwide, and the leading cause of death from a single infectious agent. Approximately 10 million new cases of tuberculosis occur annually worldwide, and 1.4 million people die of tuberculosis. Although there has been a very gradual downward trend in the past few years, TB cases are still common in developing countries in Southeast Asia and Africa [1]. In addition, although spinal caries is decreasing in developed countries, old-affected spinal caries is still common, and the number of patients with old-affected spinal caries is expected to increase in the future in regions of the developing world where the disease occurs in childhood, as in developed countries [1,2]. Spinal caries is a disease that presents with lumbar back pain, severe kyphosis, spinal cord paralysis, and intractable fistulas [3,4]. In a large study of cases from 1959–2011, it was reported that if the diagnosis is made in the pre-bone destruction stage and patients are treated with standard medications, the infection resolves in about 95% of patients with no major complications. It has been also reported that if diagnosed and treated early, neurological symptoms can be resolved without surgery in about 40% of cases [5]. However, surgery for spinal caries is actively performed, and the significance of surgery is to prevent or slow the progression of spinal cord paralysis, improve low back pain, and reduce kyphotic deformity. Surgery for spinal caries began with anterior spinal fusion (ASF) in 1934 by Ito, Tsuchiya, Asami and others, who approached the retroperitoneal space of the lumbar spine and placed a graft [6]. Also in 1956, Hodgson et al. reported 432 anterior approaches with dramatic recovery of Pott’s palsy and high bone fusion rates [7]. In recent years, with the development of instrumentation, the use of ASF alone has been decreasing, and posterior fixation alone, anterior retraction with posterior entry, fixation, and anterior-posterior merged surgery have been performed. Articles comparing ASF, posterior fixation, and combined anterior and posterior surgery have reported a reduction in kyphosis progression with combined anterior and posterior surgery [8,9,10]. Although the age of affected spinal caries has shifted from childhood to old age in developed countries, most previous reports summarize cases from childhood to young adulthood, and there are no conclusions as to which adult- to elderly-affected spinal caries patients should be treated with instrumentation [11,12,13]. In adult- and elderly-affected spinal caries, the choice of surgical procedure must take into account the possibility of decreased immunity, other pre-existing conditions, perioperative complications, kyphotic deformity, and decreased rate of fusion, and the surgical outcome needs to be reexamined. We believe that anterior scraping and reconstruction of the anterior column is important in adult and elderly patients with spinal caries, and we have basically performed ASF alone or ASF plus posterior instrumentation. In this study, we reported the surgical results of adult and elderly patients with spinal caries and investigated the rate of bone fusion and the risk factors of kyphosis progression after ASF to determine the cases requiring posterior instrumentation.

## 2. Subjects and Methods

**Figure 1:**
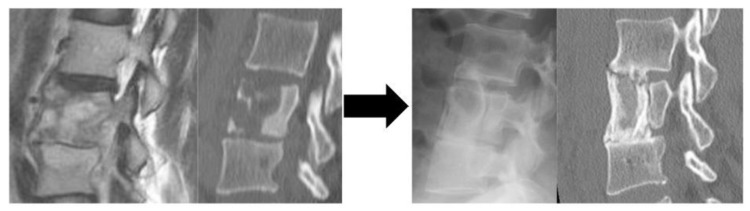
A representative of Group A patients who underwent ASF alone and did not undergo reoperation during the course of the surgery. A Preoperative CT: bone destruction at the L3 and L4 vertebrae B Immediate postoperative X-ray: Trans retroperitoneal anterior scraping and iliac bone grafting. C Postoperative CT: Bone fusing at a year postoperatively. Abbreviation: CT, computed tomography; L, lumbar; ASF, anterior spinal fusion.

**Figure 2:**
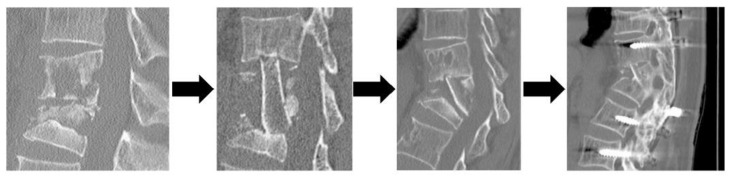
A representative of Group B patients who underwent ASF alone but underwent revision surgery. A Preoperative CT: T12, L1 vertebrae with bone destruction B Immediate postoperative X-ray: fibula grafted C Preoperative CT: postoperative kyphosis angle has progressed due to bone graft dislocation D Postoperative CT: Posterior instrumentation was added, and bone fusing was achieved 6 months later. Abbreviation: CT, computed tomography; T, thoracic; L, lumbar.

**Figure 3:**
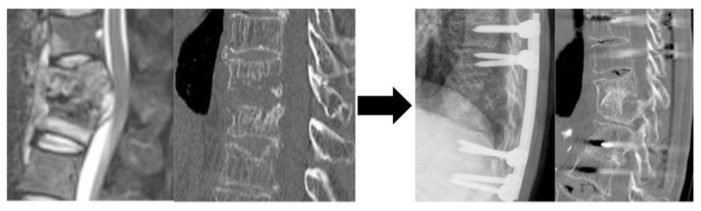
Representative case of Group C with planned ASF plus posterior instrumentation A Preoperative CT: Bone destruction at the T11 and T12 vertebrae. B Immediate postoperative X-ray: Trans-extrapleural anterior scraping and iliac bone grafting with simultaneous posterior instrumentation. C Postoperative CT: Bone fusing was achieved at 1 year postoperatively. Abbreviation: CT, computed tomography; T, thoracic; ASF, anterior spinal fusion.

From 1992 to 2019, 214 patients aged 20 years or older with spinal caries were operated only at our institution. Among them, we excluded 2 cases of cervical spine outbreaks, 10 cases of skip lesion and multiple intervertebral spine involvement where anterior scraping was not possible and only posterior fusion was performed, and 3 cases of fibula grafting to control kyphosis progression after infection and bone destruction had subsided. In addition, 134 patients with thoracic to lumbar spine outbreaks who had a follow period of more than 3 months and for whom data could be collected were included. The above subjects were divided into three groups: Group A (Figure 1), in which only ASF was performed and no revision surgery was performed during the course of the study; Group B (Figure 2), in which ASF was performed but revision surgery was performed due to the progression of local kyphosis during the course of the study; and Group C (Figure 3), which underwent planned ASF plus posterior instrumentation.

### 2.1. Indication for Surgery

The indications for surgery for spinal caries at our hospital are symptoms of neurologic deficit or severe back pain and imaging evidence of large abscess, bony cavity, dead bone, or vertebral collapse, or progressive kyphosis, despite adequate treatment with anti-tuberculosis drugs.

### 2.2. Selection of Surgical Method and Surgical Procedure

Because of the historical background of anterior fixation alone for spinal tuberculosis in children, we performed anterior fixation alone in all cases of spinal tuberculosis in adults from 1992 to 2005. However, since then, there have been many reports of anterior as well as posterior fusion being used worldwide, and since 2006, we have started to use posterior fusion in conjunction with anterior fusion, taking into account the size of the bone defect and instability, although it is the surgeon’s judgment [4]. Surgical approach was performed according to the method of Hodgson et al. [7]. 14 patients in Group B had additional posterior instrumentation with pedicle screws (PS), and one patient had a mesh cage replaced in the anterior vertebral body collapse area. Among Group C, PS was used in 18 cases, and spinous process wiring was used in one case.

### 2.3. Survey Items

The following items were investigated: the level of the affected vertebrae (thoracic T1–T10, thoracolumbar T11–L2, lumbar L3–L5, and lumbosacral L5/S) in total and in each of the three groups, the degree of paralysis according to Frankel classification before and after surgery, the use of antituberculosis drugs (four-drug combination: Rifampicin (RFP), Isoniazid (INH), Pyrazinamide (PZA), Ethambutol (EB) or RFP, INH, PZA, Streptomycin (SM), 3-drug combination: RFP, INH, EB or RFP, INH, SM, others). operative time, blood loss, type of graft bone (iliac or fibular), and number of vertebrae, for which ASF was performed [14]. In addition, imaging studies were performed to investigate the correction angle, pre- and post-operative local kyphosis angle, progression of post-operative local kyphosis angle, rate of bone fusion, and the presence rate of areas of osteosclerosis between the grafted bone and remaining vertebrae. Postoperative parameters investigated included type of orthosis (body cast, hard corset, soft corset, or none), perioperative complications, postoperative walking ability (freehand walking, T-cane walking, silver car, wheelchair), final observation period from the date of surgery. The level of the affected vertebrae should be determined as follows. When there were continuous multiple affected vertebrae, if one vertebra extended to the thoracolumbar lesion (T11–L2), it was considered to be the thoracolumbar lesion, and if one vertebra did not extend to the thoracolumbar transition (T11–L2), it was considered to be the thoracic (T1–T10) or lumbar (L3–L5) region, respectively. The bone fusion was evaluated in patients who underwent computed tomography (CT) scan after a year postoperatively, and the presence of trabeculae bone growth or bridging on Sagittal and coronal images was evaluated as presence of bone fusion [15]. Group B was evaluated by CT after the addition of posterior instrumentation and more than a year after the reoperation. The presence or absence of an osteosclerotic area between the grafted bone and the remaining vertebral body was confirmed by postoperative CT showing no osteosclerotic area on the cephalocaudal side where the graft was placed and contact between the grafted bone and the trabecular bone of the remaining vertebral body. The measured local kyphotic angle was the Cobb’s angle between the upper border of the upper normal vertebra and the lower border of the lower normal vertebra on a lateral radiograph of the lesion [16,17]. Before surgery, immediately after surgery, 3 months after surgery, 6 months after surgery, a year after surgery, and the last observation day (average 87 month) were measured. The progression of the postoperative local kyphosis angle was calculated as postoperative local kyphosis angle—immediate postoperative local kyphosis angle, respectively. In the analysis of risk factors for kyphosis progression after ASF surgery, the local postoperative 6 M kyphosis angle—the local kyphosis angle immediately after the initial surgery was used in Group A and Group B. However, for Group B patients who underwent reoperation within 6 M after initial surgery, the local kyphosis angle immediately before reoperation—the local kyphosis angle immediately after the initial surgery was calculated.

### 2.4. Analysis Contents

1. Mean and standard deviation and number of the above items in all cases
2. Number of improvements in paraplegia
3. Comparison of two groups of Group A and Group B for the above items
4. Comparison of two groups of Group A + B and Group C for the above items
5. Comparison of bone fusion rate or remaining rate of postoperative osteosclerosis area among the three groups of Group A, Group B, and Group C
6. Factors influencing kyphosis progression after ASF in Group A + B were analyzed using linear regression analysis with OLS.

### 2.5. Statistical Analysis

SPSS Statistics version 22 (IBM Corp., Armonk, NY, USA) was used for statistical analysis. Linear regression analysis was performed using the R 4.2 software (R Foundation for Statistical Computing, Vienna, Austria). For age, operative time, blood loss, number of interval spaces of ASF, kyphotic angle, progress of kyphotic angle, and final follow up were analyzed by Mann-Whitney U test, which is a nonparametric comparison. Sex differences, affected vertebra, pre and post number of Frankel classification, number of anti-tuberculosis drugs, type of bone graft, bone fusion rate and remaining rate of postoperative osteosclerosis area, post treatment, and postoperative walking ability were tested by Chi-square test. The risk factors for progression of local kyphosis angle after ASF were analyzed using linear regression analysis with the outcome being the affected level, the number of vertebrae anteriorly fixed, and the preoperative local kyphosis angle after adjustment for age, type of grafted bone, presence or absence of areas of osteosclerosis between the grafted bone and remaining vertebrae, and type of orthosis. For the affected level, the thoracolumbar transition was used as a reference to compare the thoracic, lumbar, and lumbosacral, respectively in Group A + B. The same conditions were used to compare the progression of kyphosis angle to the affected level in Group A + B, when Group C was reference. A *p* value < 0.05 was considered to show a statistically significant difference.

## 3. Results

### 3.1. Pre- and Postoperative Status of All Patients in this Study

Mean age at surgery: 63 years, 78 males, 56 females, mean observation period: 2.7 years. The thoracolumbar transition was the most common site of affected level. Both preoperatively and postoperatively, Frankel classification D and E were common. Surgery improved from A to B in one case, from B to C in one case, from C to D in six cases, and from D to E in 22 cases. The degree of postoperative paralysis of the lower extremities did not differ significantly between groups. The iliac bone was mainly used as the graft bone. The number of anteriorly fixed vertebrae was mainly one and two, with an average of 1.6 vertebrae. The majority of orthoses were hard corsets. There were 36 perioperative complications: three cases of thoracic duct injury, a case of postoperative paralysis, 11 cases of graft bone dislocation, a case of bacterial superficial infection, and two cases of bacterial deep infection. There were three cases of pneumonia, two cases of urinary tract infection, a case of gastric ulcer, a case of duodenal ulcer, a case of intestinal perforation, two cases of intestinal obstruction, two cases of cerebral infarction, a case of transient ischemic injury, two cases of bedsore, and three cases of anorexia. After discharge from the hospital, most patients walked alone or with T-cane. Imaging data showed that the mean correction angle was 4.6, and local kyphosis had progressed in the postoperative course (Table 1).

### 3.2. Comparison among the Three Groups in Various Pre- and Post-Operative Factors

Group A consisted of 100 patients, Group B consisted of 15 patients (mean date of reoperation from initial operation 11 M), and Group C consisted of 19 patients. There were no significant differences in all items between Group A and Group B (Table 2). In the comparison between Group A + B and Group C, surgery time is long and the progression of postoperative local kyphotic angle after postoperative 6 M were significantly smaller in Group C (Table 3).

### 3.3. Bone Fusion Rates for Anterior Fusion and Anterior-Posterior Surgery

The bone union rate of 119 cases, which had CT scans taken more than a year later, and the date of CT scan used to evaluate bone fusion was two years from the average surgery date, was 83.2%. Of 88 cases in Group A, there were four cases in which both the cephalad and caudal sides of the graft did not fuse, seven cases in which the cephalad side of the graft fused but the caudal side did not fuse, and a case in which the caudal side of the graft fused but the cephalad side did not fuse, resulting in a bone fusion rate of 86.2%. In Group B, out of 15 cases, the head and tail sides of the graft did not fuse together in 1 case, the head side of the graft fused but the tail side did not fuse in 1 case, and the tail side of the graft fused but the head side did not fuse in 1 case, resulting in a bone fusion rate of 80%. In Group C, out of 17 cases, the cephalic side of the graft was fused but the caudal side was not fused in 2 cases, and the caudal side of the graft was fused but the cephalic side was not fused in 2 cases, resulting in 70.6%. There was no significant difference between the three groups. The ratio of an osteosclerotic area between the grafted bone and the remaining vertebral body was 6.8% overall, and the percentages for each group were 5% for Group A, 26.6% for Group B, and 10.5% for Group C, respectively. In the comparison of the three groups, Group B had a significantly higher rate.

### 3.4. Postoperative Progression of Kyphosis Deformity in Anterior Fusion and Anterior-Posterior Surgery

In Group A + B, only the affected level was an influential factor in the progression of local kyphosis angle after ASF, whereas the preoperative kyphosis angle and the number of fixed interspaces were not. With regard to the affected level, postoperative kyphosis did not progress in the thoracic and lumbar regions relative to the thoracolumbar transition (Table 4). In contrast to Group C, the kyphosis angle in Group A + B progressed at the thoracolumbar transition, but not at the thoracic or lumbar spine. For lumbosacral lesions, the difference was marginally significant at *p* = 0.06 (Table 5).

## 4. Discussions

### 4.1. Patient Background and Course of Treatment in this Study

The majority of preoperative Frankel classification were D or E. This may be due to the fact that the spinal cord injury appeared before the patient developed severe kyphosis, or that the patient was diagnosed with spinal caries early and was treated with antibiotics before paralysis appeared. The kyphosis angle after ASF was more advanced than that reported in previous reports where the average age of patients was in the 30s, but the age at surgery at our institution was older than that reported in previous reports, which may have been influenced by age-related bone quality loss [13,18]. Postoperative walking ability improved from walking with T cane to walking with free hand in most of the cases except for the case where the paralysis was severe before the surgery and did not improve, and the fact that the paralysis worsened after the surgery in only one case suggests that the surgery at our hospital has achieved stable results.

### 4.2. Spinal Cord Paralysis in Spinal Tuberculosis

In children with spinal tuberculosis, an abscess called a cold abscess causes spinal paralysis, known as Pott’s paralysis. Fortunately, most cases of paralysis of the lower extremities at the time of initial diagnosis at our facility were either absent or mild. This may be due to the fact that there is a specialized pulmonary tuberculosis hospital in the neighborhood of our facility, and patients were referred to this hospital after they were treated early with anti-tuberculosis therapy. On the other hand, patients who were referred to our facility after so-called “doctor’s delay” were found to have severe paralysis of the lower limbs. Needless to say, the decreasing number of spinal TB cases and the aging of the population are factors, but orthopedic surgeons still need to know the basics of spinal TB and always be suspicious of spinal caries. Spinal TB is characterized by lumbar back pain and paralysis of the lower extremities, which is common in the compromised host. Slight fever and slightly increased C-reactive protein are present. Osteolytic bone destruction is seen, sometimes involving multiple intervertebral spaces; Magnetic resonance imaging (MRI) shows low intensity on T1-weighted images, high intensity on T2-weighted images, and rim enhancement on contrast enhancement MRI. In such cases, the diagnosis can be made by testing for Mycobacterium tuberculosis Polymerase chain reaction and tuberculosis Interferon-γ [19,20]. If surgery is performed with a clear understanding of these basic principles, the frequency of lower limb paralysis can be reduced. The degree of paralysis of the lower extremities due to surgery appears to be good. In particular, many patients go from C to D and D to E. In addition, there were cases in which A and B patients also improved, suggesting that aggressive surgical treatment is desirable for patients with neurological symptoms. The degree of postoperative paralysis of the lower extremities was comparable between the groups, and thus was not considered to influence the choice of surgical technique.

### 4.3. Importance and Complications of ASF in Spinal Caries

It has been considered best to expand the lesion anteriorly under direct vision rather than posteriorly, because the lesion can be adequately dissected, there is less possibility of damage to vital organs because there is no blind manipulation, and allograft bone can be filled and grafted as a primary treatment for spinal caries in any region [21]. The bone histology of tuberculous spondylitis also suggests the importance of anterior retraction. Izawa et al. reported that the histopathology of spinal caries showed bone destruction due to activation of the receptor activator of nuclear factor kappa-Β (RANK)_RANK ligand system, but the expression of osteoprotegerin, which is a mechanism to inhibit bone resorption, was not significantly different from that of the control group, and the expression of osteocalcin, a marker of bone formation, was poor. They reported that the results were consistent with the clinical picture of spinal tuberculosis, which shows bone destruction with bone resorption predominance [22]. These findings suggest that firm scraping of the lesion is important not only for stopping the progression of paralysis but also for bone fusion. Although ASF in spinal caries is a safe procedure, there are some cautions. First of all, since it is difficult to identify the tissue due to the cold abscess, a contrast-enhanced CT scan in the lateral supine position must be performed before the procedure, and attention must be paid to large vessels, ureter, thoracic duct, and nerve damage. It is also advisable to use a nerve stimulator and perform detailed stimulation when it is difficult to differentiate between nerves and other tissues. Fortunately, there were no cases of vascular or ureteral injuries among the complications at our institution, but there were two cases of thoracic duct injuries. They occurred predominantly at the thoracolumbar transition, and were thought to be due to the anatomical running of the thoracic duct [23]. Other complications were not unique to this disease, as they often occur in elderly patients even after surgery for other diseases.

### 4.4. Determining the Extent of the Anterior Scraping

While bone sequestrum is easily visible on CT, it is not possible to distinguish between bone fragments consisting of necrotic bone, residual viable bone, or matrix calcification. MRI may also be able to distinguish between truly avascular ossicles surrounded by necrotic tissue and normal bone with residual vascularity surrounded by vascularized lesion tissue, but it is difficult to accurately determine the extent of the scraping preoperatively at this time [24]. We check the trabecular bone stiffness intraoperatively to avoid increasing the deficit space by excessive curettage, and try to avoid increasing the number of intervertebral spaces required for ASF by removing soft areas and leaving hard areas as much as possible. Although osteosclerotic images often accompany the surrounding decayed bone, removing the osteosclerotic area as much as possible on the cephalocaudal side where the bone graft is implanted is an important condition for bone fusion. It is important to understand the osteosclerotic area by preoperative CT because it may be perceived as normal bone by intraoperative feel and may be left behind.

### 4.5. Methods for Determining Bone Fusion and the Rate of Bone Fusion after Spinal Caries Surgery

The hallmark of bone fusion in caries is replacement by fibrous connective, cartilage connective tissue, and new bone formation [25]. As for the method of determining bone fusion, there are several reports that have evaluated it by X-ray and CT [15,26,27,28]. In this study, since CT was performed in the majority of patients more than 1 year after surgery, trabeculae bone growth between the graft and residual vertebrae and bridging between the residual vertebrae were evaluated as bone fusion, mimicking the report by John et al. [15]. The results showed that the overall rate of bone fusion was 83.2%, and there was no difference between all groups. In the present series, the osteosclerotic area was removed in 93.2% of cases, but the bone fusion rate was also good, suggesting that sufficient bone fusion can be achieved as long as the osteosclerotic area on CT is removed. However, there were cases where the grafted bone fractured, crushed or dislocated during the course of the fusing process, resulting in progressive kyphosis or reoperation. Therefore, we could not be optimistic even though the rate of bone fusion was high, and we could not judge the surgical methods according to the rate of bone fusion.

### 4.6. Risk Factors for Kyphosis Progression after ASF and Cases Requiring Posterior Instrumentation

It has been reported that the average correction rate of kyphosis angle in ASF is 62.5%, but the correction loss to 24.1% in the postoperative course [29]. In the present study, some of the patients developed graft dislocation and rapid local kyphosis angle after ASF, but after the addition of posterior instrumentation, the patients had a stable course. In addition, in patients who underwent planned ASF and posterior instrumentation, the graft did not dislodge and the progression of kyphosis was controlled. Therefore, we believe that the use of posterior instrumentation should be aggressively pursued. On the other hand, for elderly and immunocompromised patients with spinal caries, it is necessary to reduce complications as minimally invasive as possible. In the case of combined anterior and posterior surgery, the use of posterior instrumentation for all patients is excessive because the operation time is long, infection has been observed in the posterior approach in previous papers and this series, and implant failure is expected to occur in osteoporotic patients [30]. It is important to limit the use of posterior instrumentation and use it appropriately. As to the question of when to use posterior instrumentation, risk factors for kyphosis progression after ASF were analyzed using linear regression analysis. In this series, there were only a few cases of residual osteosclerotic areas after surgery, so they cannot be discussed per se. Since the residual rate was high in Group B, which was a revision surgery case, it was added in a confounding factor to eliminate the effect. The results showed that the number of fixed vertebrae was not a risk factor. The reason for this may be that at our institution, as mentioned above, the extent of ASF is limited to one or two intervertebral spaces. When comparing the progression of postoperative kyphosis angle in the four groups of affected vertebral body levels, there was less kyphosis progression in the thoracic and lumbar regions, and kyphosis progression in the thoracolumbar transition when compared to Group C. These results suggest that posterior instrumentation should be added systematically when the affected vertebra is at the thoracolumbar transition, and that ASF alone in the thoracic and lumbar regions may result in less progression of the kyphoscoliosis angle. No conclusion can be drawn from this analysis about lumbosacral lesions. Whether posterior instrumentation is necessary needs to be examined again with more cases.

## 5. Conclusions

Spinal tuberculosis should be diagnosed early and patients with neurological symptoms should be treated surgically. The improvement of paralysis and fusion rate of ASF in spinal caries was good. The progression of the local kyphosis angle after ASF was less in the thoracic and lumbar regions. On the other hand, in cases of caries at the thoracolumbar transition where ASF alone was performed, postoperative kyphosis progressed, suggesting the need for posterior instrumentation in such cases.

### Limitations

First of all, this study was retrospective, with a small number of cases and a wide range of cases collected in the last observation period. In addition, the date of bed release could not be investigated from the past records. Furthermore, the clinical output after surgery could only be assessed on a 4-point scale of gait status, and clinical outcomes could not be linked to the progression of the kyphosis angle after surgery. For this reason, it was thought necessary to study cases and conduct prospective studies.

## Figures and Tables

**Table 1:** Preoperative and Postoperative Characteristics of All Patients.

N		134
Age		63 ± 17
Male/Female		78/56
Affected vertebra	Thoracic (T1–T10)	34
	Thoracolumbar (T11–L2)	55
	Lumbar (L3–L5)	38
	Lumbosacral (L5/S)	7
Preoperative Frankel classification	A	4
	B	2
	C	17
	D	44
	E	66
Postoperative Frankel classification	A	3
	B	3
	C	10
	D	28
	E	88
Anti-TB drug	4 types	87
	3 types	43
	Other	4
Operation time (min)		244 ± 72
Blood loss (mL)		573 ± 509
Bone graft	Iliac bone	104
	Fibula bone	30
Number of intervertebral space of ASF		1.6 ± 0.8
Correction angle (°)		4.5 ± 7
Kyphotic angle (°)	Preoperative	6.6 ± 14.6
	Immediately after op	2 ± 15.3
	Postoperative 3 M	7 ± 16.6
	Postoperative 6 M	8.4 ± 17.6
	Postoperative 1 Y	9.5 ± 16.7
	Final follow-up	10.4 ± 17.3
Progress of kyphotic angle (°)	Postoperative 3 M—immediately after op	4.9 ± 7.9
	Postoperative 6 M—immediately after op	7.3 ± 8.5
	Postoperative 1 Y—immediately after op	8 ± 8.9
	Postoperative final follow-up—immediately after op	8.7 ± 9.3
Bone fusion rate (%)		83.2
Remaining osteosclerotic areas after op (%)		6.8
Posttreatment	Body cast	16
	Hard corset	111
	Soft corset	13
	None	6
Complications		36
Postoperative walking ability	Free hand	55
	T-cane	48
	Silver car	17
	Wheelchair	10
Final follow-up year (years)		2.7 ± 2.3

The mean age at the time of surgery was 66 years, which was higher than the mean age, but the postoperative local kyphosis angle had progressed. Abbreviation: T, thoracic; L, lumbar; S, sacrum; M, month; Y, year; ASF, anterior spinal fusion; OP, operation.

**Table 2:** Comparison of Preoperative and Postoperative Characteristics of Patients in Groups A and B.

		A	B	*p* Value
N		100	15	
Age		61 ± 16	67 ± 10	n.s.
Male/Female		62/38	6/9	n.s.
Affected vertebra	Thoracic (T1–T10)	27	2	n.s.
	Thoracolumbar (T11–L2)	41	6	n.s.
	Lumbar (L3–L5)	28	5	n.s.
	Lumbosacral (L5/S)	4	2	n.s.
Preoperative Frankel classification	A	2	1	n.s.
	B	2	0	n.s.
	C	17	0	n.s.
	D	29	6	n.s.
	E	50	8	n.s.
Postoperative Frankel classification	A	2	1	n.s.
	B	2	0	n.s.
	C	10	0	n.s.
	D	19	3	n.s.
	E	67	11	n.s.
Anti-TB drug	4 types	66	8	n.s.
	3 types	31	6	n.s.
	Other	2	0	n.s.
Operation time (min)		224 ± 62	236 ± 50	n.s.
Blood loss (mL)		625 ± 584	359 ± 255	n.s.
Bone graft	Iliac bone	77	9	n.s.
	Fibula bone	23	6	n.s.
Number of intervertebral space of ASF		1.6 ± 0.7	1.8 ± 1	n.s.
Correction angle (°)		5.2 ± 7.1	4.3 ± 6.4	n.s.
Kyphotic angle (°)	Preoperative	6.44 ± 14.8	10.3 ± 16.1	n.s.
	Immediately after op	1.3 ± 15.2	5.9 ± 14.5	n.s.
	Postoperative 3 M	5.8 ± 16.1	15.9 ± 18	n.s.
	Postoperative 6 M	8.2 ± 16.8	16.9 ± 21	n.s.
	Postoperative 1 Y	10.2 ± 16.4	13.2 ± 17.8	n.s.
	Final follow-up	10.2 ± 16.7	17.2 ± 19.5	n.s.
Progress of kyphotic angle (°)	Postoperative 3 M—immediately after op	4.7 ± 8	8.5 ± 9.8	n.s.
	Postoperative 6 M—immediately after op	7.8 ± 8.2	10.9 ± 10.5	n.s.
	Postoperative 1 Y—immediately after op	9.6 ± 8.8	5.5 ± 10.2	n.s.
	Final follow-up—immediately after op	9.5 ± 9.5	10.7 ± 9.6	n.s.
Posttreatment	Body cast	16	0	n.s.
	Hard corset	83	14	n.s.
	Soft corset	13	0	n.s.
	None	5	0	n.s.
Complications		29	3	n.s.
Postoperative walking ability	Free hand	39	8	n.s.
	T-cane	38	5	n.s.
	Silver car	14	1	n.s.
	Wheelchair	9	1	n.s.
Final follow-up year (years)		2.6 ± 2.2	3.3 ± 2.9	n.s.

There were no significant differences in all items between Group A and Group B. Abbreviation: T, thoracic; L, lumbar; S, sacrum; M, month; Y, year; ASF, anterior spinal fusion; OP, operation; n.s., not significant.

**Table 3:** Comparison of Preoperative and Postoperative Characteristics of Patients in the Combined Group A + B and Group C.

		A + B	C	*p* Value
N		115	19	
Age		61.8 ± 16.2	69.8 ± 17	n.s.
Male/Female		68/47	10/9	n.s.
Affected vertebra	Thoracic (T1–T10)	29	5	n.s.
	Thoracolumbar (T11–L2)	47	8	n.s.
	Lumbar (L3–L5)	33	5	n.s.
	Lumbosacral (L5/S)	6	1	n.s.
Preoperative Frankel classification	A	3	1	n.s.
	B	2	0	n.s.
	C	17	0	n.s.
	D	35	9	n.s.
	E	58	8	n.s.
Postoperative Frankel classification	A	3	0	n.s.
	B	2	1	n.s.
	C	10	0	n.s.
	D	22	6	n.s.
	E	78	10	n.s.
Anti-TB drug	4 types	74	13	n.s.
	3 types	37	5	n.s.
	Other	3	1	n.s.
Operation time (min)		226 ± 60	310 ± 77	<0.01
Blood loss (mL)		590 ± 558	525 ± 368	n.s.
Bone graft	Iliac bone	86	18	n.s.
	Fibula bone	29	1	n.s.
Number of intervertebral space of ASF		1.7 ± 0.8	1.6 ± 0.8	n.s.
Correction angle (°)		5 ± 7	1.6 ± 6.7	n.s.
Kyphotic angle (°)	Preoperative	6.9 ± 15	4.4 ± 12.2	n.s.
	Immediately after op	1.9 ± 15.1	2.8 ± 16.2	n.s.
	Postoperative 3 M	7.1 ± 16.7	6.1 ± 16.2	n.s.
	Postoperative 6 M	9.4 ± 17.7	1.9 ± 15.4	n.s.
	Postoperative 1 Y	10.6 ± 16.6	3.2 ± 15.3	n.s.
	Postoperative final follow-up	11.1 ± 17.2	6.1 ± 16.8	n.s.
Progress of kyphotic angle (°)	Postoperative 3 M—immediately after op	5 ± 8.1	2.9 ± 3.7	n.s.
	Postoperative 6 M—immediately after operation	8.2 ± 8.7	1.2 ± 4.7	<0.01
	Postoperative 1 Y—immediately after op	9.3 ± 8.8	2.5 ± 4	<0.01
	Postoperative final follow-up—immediately after op	9.4 ± 9.7	3.2 ± 4.5	<0.01
Posttreatment	Body cast	16	0	n.s.
	Hard corset	97	14	n.s.
	Soft corset	13	0	n.s.
	None	5	1	n.s.
Complications		32	4	n.s.
Postoperative walking ability	Free hand	47	8	n.s.
	T-cane	43	5	n.s.
	Silver car	15	2	n.s.
	Wheelchair	10	0	n.s.
Final follow-up year (years)		2.8 ± 2.7	2.5 ± 2.1	n.s.

Compared to Group A + B, Group C had less advanced kyphosis postoperatively. Abbreviation: T, thoracic; L, lumbar; S, sacrum; M, month; Y, year; ASF, anterior spinal fusion; OP, operation; n.s., not significant.

**Table 4:** Linear regression analysis of factors associated with kyphosis progression after ASF when the thoracolumbar region was used as a reference.

	Est.	2.50%	97.50%	t Value	*p* Value
Affected vertebral body level: Thoracic	−4.1	−8.25	0.04	−1.96	0.04 *
: Lumbar	−4.21	−8.16	−0.25	−2.11	0.04 *
: Lumbosacral	0.55	−6.79	7.89	0.15	0.88
Age	0.01	−0.1	0.11	0.15	0.88
Remaining osteosclerotic area after ASF	−5.96	−11.51	−0.41	−2.13	0.04
Types of grafted bone	2.27	−1.6	6.13	1.16	0.25
Soft brace	−4.52	−13.66	4.63	−0.98	0.33
Hard brace	−3.65	−11.44	4.14	−0.93	0.35
Body cast	−6.75	−15.3	1.8	−1.57	0.12

The thoracolumbar affected vertebrae was significantly more likely to cause kyphosis than the thoracic or lumbar spine in Group A + B. When *p* < 0.05 and there is a significant difference, * is appended to the upper right of the number. Abbreviation: ASF, anterior spinal fusion.

**Table 5:** Analysis of factors associated with kyphosis progression after ASF when kyphosis progression in Group C was used for reference.

	Est.	2.50%	97.50%	t Value	*p* Value
Affected vertebral body level: Thoracic	3.2	−2.41	8.8	1.13	0.26
: Thoracolumbar	7.35	2.16	12.54	2.8	0.01 **
: Lumbar	3.05	−2.4	8.51	1.11	0.27
: Lumbosacral	8.04	−0.23	16.31	1.93	0.06
Age	0	−0.09	0.09	0.08	0.93
Remaining osteosclerotic area after ASF	−4.26	−9.1	0.59	−1.74	0.08
Types of grafted bone	2.36	−1.22	5.95	1.31	0.19
No brace	4.74	−13.27	22.74	0.52	0.6
Soft brace	1.22	−15.09	17.53	0.15	0.88
Hard brace	1.19	−15.33	17.7	0.14	0.89
Body cast	−1.9	−18.91	15.07	−0.22	0.82

The progression of postoperative kyphosis angle in Group C was diminished compared with that of the thoracolumbar affected vertebrae in Group A + B. When *p* < 0.01 and there is a significant difference, ** is appended to the upper right of the number. Abbreviation: ASF, anterior spinal fusion.

## Data Availability

The deidentified participant data will be shared by the corresponding author upon reasonable request.
